# Case Report: Complete MRI-confirmed remission of presumptive meningoencephalitis of unknown origin in a Chihuahua using a steroid-free combination of leflunomide and mycophenolate mofetil

**DOI:** 10.3389/fvets.2026.1750629

**Published:** 2026-02-19

**Authors:** Sung Su Park

**Affiliations:** IU Animal Medical Center, Seongnam-si, Republic of Korea

**Keywords:** canine MUO, immunosuppression, leflunomide, MRI-confirmed remission, mycophenolate mofetil, steroid-free therapy

## Abstract

Meningoencephalitis of unknown origin (MUO) is an idiopathic inflammatory disorder of the canine central nervous system predominantly affecting small-breed dogs. Standard treatment typically involves glucocorticoids; however, chronic steroid exposure carries substantial risk for adverse effects and may compromise long-term management. A 9-year-old, 2.5 kg female Chihuahua presented with 1 week of rightward circling, head tilt, and cervical stiffness. MRI revealed multifocal intra-axial hyperintensities involving the cerebellum, brainstem, and medulla with associated meningeal enhancement. CSF cytology demonstrated moderate lymphocytic pleocytosis with elevated protein. A steroid-free immunosuppressive protocol using leflunomide and mycophenolate mofetil was initiated. Neurologic signs progressively improved, and repeat MRI at 12 months documented complete resolution of inflammatory lesions. Both medications were continued for 2 years, during which serial hematologic and biochemical monitoring remained within normal limits. No adverse events occurred. This case provides, to the authors’ knowledge, the first documented MRI-confirmed remission of MUO achieved with exclusive steroid-free immunosuppression. Relevant literature is reviewed to contextualize the immunopathogenesis of MUO and considerations for steroid-sparing strategies.

## Introduction

Meningoencephalitis of unknown origin (MUO) refers to a spectrum of idiopathic, presumed immune-mediated inflammatory diseases of the canine central nervous system, including granulomatous meningoencephalomyelitis, necrotizing leukoencephalitis, and necrotizing meningoencephalitis (1–4). These conditions are disproportionately represented in small-breed adult dogs and often present with acute or sub-acute multifocal neurological deficits. Typical signs include ataxia, circling, head tilt, proprioceptive deficits, seizures, and cervical pain (1, 2). Because histopathologic confirmation is rarely feasible *in vivo*, diagnosis is typically presumptive based on characteristic MRI patterns, cerebrospinal fluid (CSF) abnormalities, and exclusion of infectious triggers (2–4).

Glucocorticoids have been the foundation of MUO management since the earliest large case series, due to their ability to dampen inflammation rapidly (5, 6). However, long-term steroid use is associated with a significant burden of adverse effects (hepatopathy, gastrointestinal ulceration, muscle atrophy, insulin resistance, polyphagia, immunosuppression), which may substantially impact small-breed dogs under prolonged treatment (6, 7). These concerns have prompted increasing interest in adjunctive immunosuppressive agents and steroid-sparing strategies.

In canine MUO treatment, adjunctive immunosuppressants—including cytarabine, lomustine, cyclosporine, mycophenolate mofetil (MMF), and leflunomide—have been explored to improve survival and minimize steroid dosage (3, 8–10). MMF functions via inhibition of inosine monophosphate dehydrogenase, reducing lymphocyte proliferation, whereas leflunomide targets pyrimidine synthesis and inhibits both T- and B-cell activation and cytokine expression (11, 12). Despite these theoretical advantages, most published reports continue to describe glucocorticoids as the core of therapy, and evidence supporting exclusive use of MMF and leflunomide without steroids remains limited.

To the authors’ knowledge, no previous reports have documented MRI-confirmed complete remission achieved solely through steroid-free immunosuppression. The present case aims to fill that gap, documenting detailed imaging, CSF, clinical, and laboratory follow-up over a two-year period, while also providing a contextual literature review of MUO immunopathogenesis, treatment paradigms and steroid-sparing considerations.

## Case description

**Figure 1:**
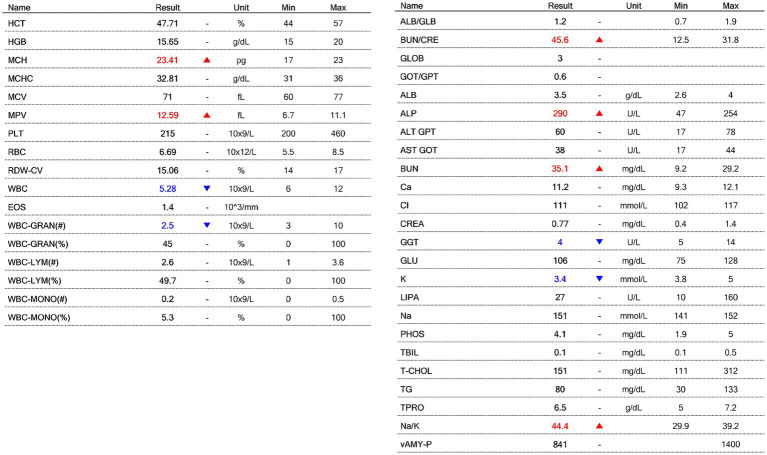
Baseline hematologic and serum biochemical results obtained at presentation, prior to initiation of immunosuppressive therapy.

A 9-year-old, 2.5-kg neutered female Chihuahua was evaluated in October 2023 for a one-week history of rightward circling, right-sided head tilt, and cervical rigidity. The neurological signs progressed sub-acutely without seizures or systemic illness. Neurological examination suggested involvement of the right forebrain and caudal fossa. Baseline complete blood count (CBC; BCC3000B VET ABC™, Vetcom, Korea) and serum biochemical analysis (DryChem NX600™, Fujifilm Corp., Tokyo, Japan) were within reference intervals (Figure 1), supporting the absence of systemic or infectious disease and confirming suitability for immunosuppressive therapy.

Magnetic resonance imaging (MRI) revealed multifocal intra-axial inflammatory lesions involving the cerebellum, pons, medulla, and forebrain. The affected regions exhibited poorly marginated T2-weighted hyperintensity consistent with vasogenic and cytotoxic edema typically observed in MUO (1–4). Corresponding hyperintensity on fluid-attenuated inversion recovery (FLAIR) sequences supported true parenchymal involvement rather than cerebrospinal fluid (CSF) signal contamination, while subtle T1-weighted hypointensity was consistent with non-necrotizing inflammatory encephalitis. Following gadolinium administration, patchy parenchymal and mild leptomeningeal contrast enhancement indicated disruption of the blood–brain barrier, a characteristic imaging feature of MUO and an important discriminator from congenital or age-related changes (2–4). Minimal mass effect was present, and no hemorrhage, necrosis, or discrete mass lesions were identified.

**Figure 2:**
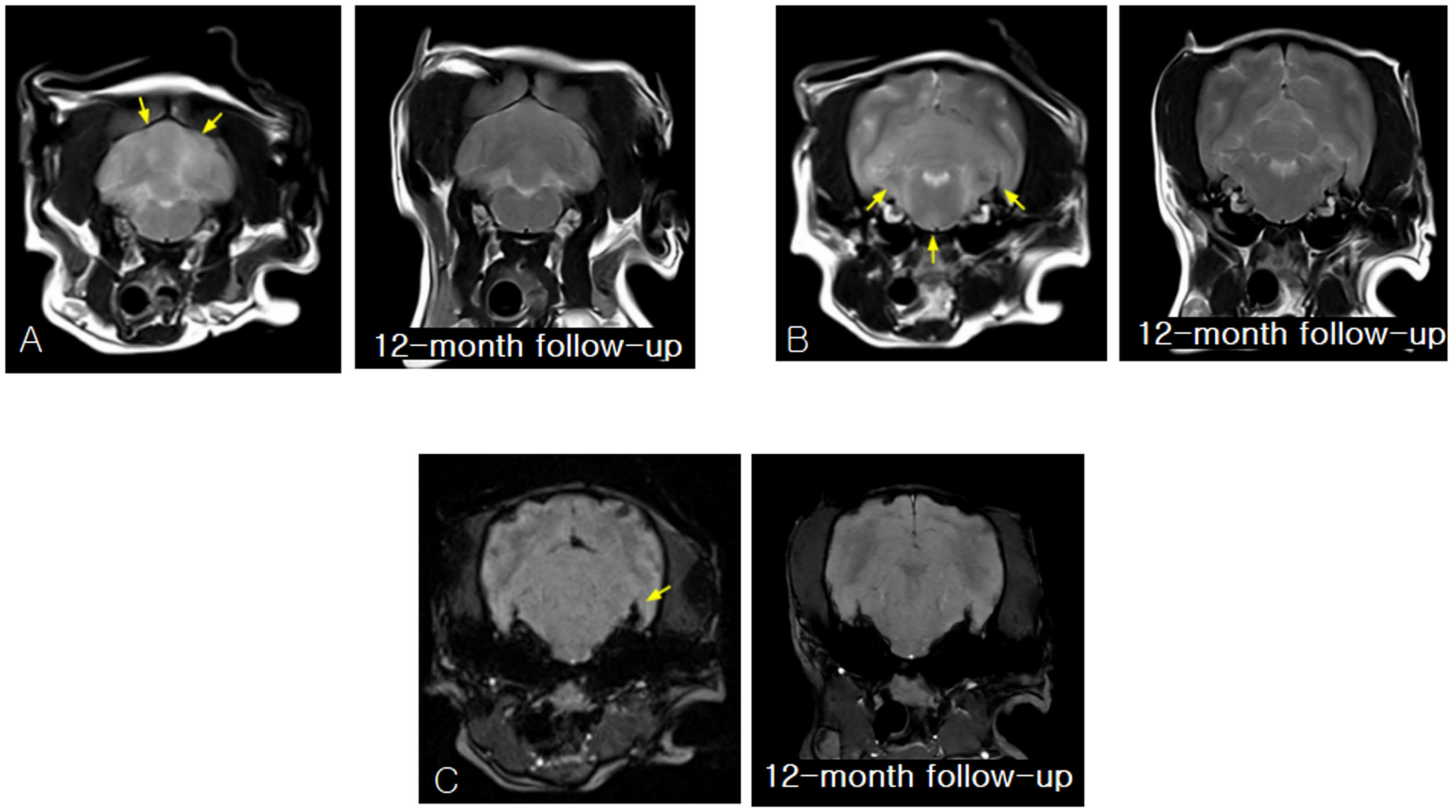
Initial and 12-month follow-up magnetic resonance imaging (MRI) findings obtained after initiation of steroid-free immunosuppressive therapy, demonstrating resolution of all previously identified inflammatory lesions. **(A)** Baseline transverse T2-weighted image showing bilateral, poorly marginated hyperintensity within the cerebellar hemispheres, consistent with diffuse intra-parenchymal inflammatory change. At the 12-month follow-up, a transverse T2-weighted image obtained at the same anatomical level demonstrates normalization of cerebellar parenchymal signal intensity, with resolution of the previously observed T2 hyperintensities and restoration of normal gray–white matter distinction. **(B)** Baseline transverse T2-weighted image demonstrating ill-defined, bilateral hyperintense lesions involving the pons and medulla, consistent with non-mass-like inflammatory infiltration of the caudal brainstem. At the 12-month follow-up, a transverse T2-weighted image obtained at the same anatomical level shows resolution of the previously identified bilateral T2 hyperintense lesions, with restoration of a uniform and symmetrical brainstem parenchymal signal and no evidence of residual inflammation. **(C)** Baseline susceptibility-weighted imaging (SWI) revealing an asymmetric hypointense focus within the left pontine region, suggestive of localized susceptibility changes compatible with active inflammatory disease. At the 12-month follow-up, SWI obtained at the same level demonstrates disappearance of the previously observed hypointense focus, with no residual susceptibility artifact, indicating resolution of the inflammatory-associated signal change.

**Figure 3:**
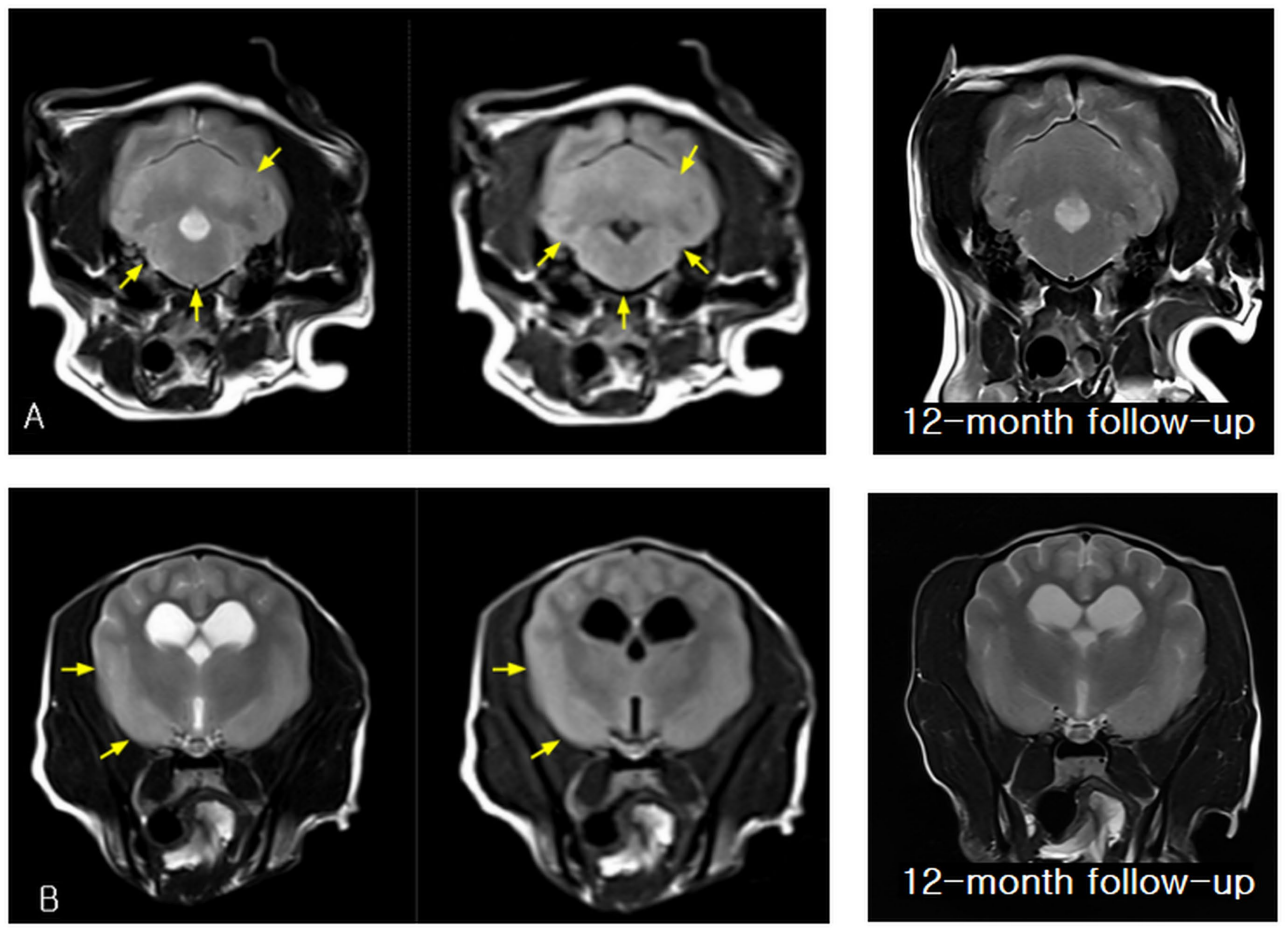
Baseline and 12-month follow-up magnetic resonance imaging (MRI) findings obtained after initiation of steroid-free immunosuppressive therapy, demonstrating resolution of inflammatory lesions involving the brainstem and forebrain. **(A)** Baseline transverse T2-weighted and fluid-attenuated inversion recovery (FLAIR) images showing bilateral, poorly marginated hyperintensity involving the pons and medulla, consistent with inflammatory parenchymal involvement of the caudal brainstem. At the 12-month follow-up, a transverse T2-weighted image obtained at the same anatomical level demonstrates normalization of parenchymal signal intensity within the pons and medulla, with no evidence of residual abnormal signal. **(B)** Baseline transverse T2-weighted and FLAIR images of the forebrain demonstrating mild right frontal cortical and subcortical hyperintensity, indicating rostral extension of the inflammatory process. At the 12-month follow-up, a transverse T2-weighted image of the forebrain shows normal cortical and subcortical signal intensity, with resolution of the previously observed right frontal abnormalities and no abnormal signal intensity or structural distortion.

Baseline and 12-month follow-up MRI findings are presented together in Figures 2, 3 to illustrate the temporal evolution and subsequent resolution of these inflammatory lesions.

Concurrent congenital anomalies, including an arachnoid cyst and syringohydromyelia, were also identified but were considered incidental, as they lacked surrounding gliosis, were inconsistent with the acute onset of neurological signs, and remained unchanged on follow-up imaging. Similar incidental findings have been reported previously in small-breed dogs without an established association with MUO onset (3).

Cerebrospinal fluid analysis revealed moderate lymphocytic pleocytosis (total nucleated cell count approximately 140 cells/μL, with 80–90% lymphocytes) and increased protein concentration. This cytologic profile is consistent with previously described CSF findings in MUO, which are typically lymphocytic or mixed lymphocytic–monocytic (1, 3, 4). No organisms suggestive of infectious disease were identified, and polymerase chain reaction (PCR) testing for canine distemper virus, *Toxoplasma gondii*, *Neospora caninum*, and *Bartonella* spp. was negative. Collectively, the MRI and CSF findings supported a presumptive diagnosis of MUO in accordance with established diagnostic criteria (1–4).

Because of the dog’s small body size, the anticipated need for prolonged immunosuppressive therapy, and concerns regarding chronic glucocorticoid-associated adverse effects, a steroid-free immunosuppressive protocol was initiated. Conventional MUO management relies heavily on glucocorticoids; however, long-term steroid exposure is associated with hepatopathy, gastrointestinal ulceration, muscle atrophy, insulin resistance, and immune suppression, particularly in small-breed dogs (6, 7). To mitigate these risks, leflunomide (5 mg/kg, once daily) and mycophenolate mofetil (10 mg/kg, once daily) were selected. Mycophenolate mofetil inhibits T- and B-lymphocyte proliferation via inhibition of inosine monophosphate dehydrogenase, whereas leflunomide suppresses pyrimidine synthesis and cytokine production (11, 12). Previous studies have evaluated these agents primarily in combination with glucocorticoids (8–10), and evidence supporting their exclusive use as a steroid-free regimen remains limited.

Neurological improvement progressed steadily, with resolution of circling behavior by week six, improvement of cervical rigidity by month three, and normalization of proprioceptive deficits by month six. No seizure activity was observed during the treatment period. Follow-up MRI performed 12 months after initiation of therapy demonstrated complete resolution of the previously identified inflammatory lesions, including normalization of T2- and FLAIR-weighted signal abnormalities, disappearance of susceptibility changes on susceptibility-weighted imaging, and absence of abnormal contrast enhancement (Figures 2, 3). This degree of radiologic resolution is notable, as residual structural abnormalities frequently persist in dogs with MUO despite clinical improvement (3, 13).

**Figure 4:**
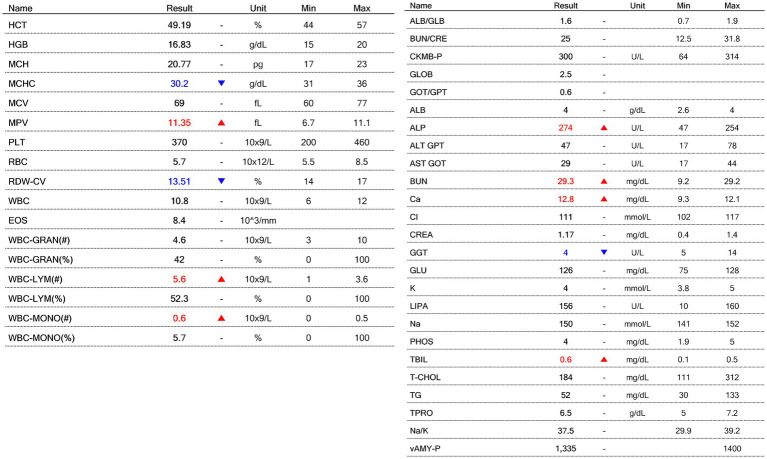
Hematologic and serum biochemical results obtained 24 months after initiation of steroid-free immunosuppressive therapy.

Because relapse commonly occurs within 6–12 months—even after apparent remission—both medications were continued for two full years, in accordance with long-term management recommendations (3, 13, 14). Serial hematologic and biochemical monitoring remained within normal limits with no evidence of bone marrow suppression, hepatotoxicity, nephrotoxicity, or metabolic disturbance (Figure 4). The complete absence of adverse events over 24 months is clinically meaningful, as many MUO treatment failures result from drug-related complications rather than disease progression (7, 13). At the 24-month evaluation, the dog remained neurologically normal, with full restoration of behavior and activity. The combination of clinical remission, MRI-confirmed resolution, and sustained laboratory stability indicates durable control of MUO using a fully steroid-free regimen—an outcome not previously documented in the veterinary literature.

## Discussion

This case describes a Chihuahua with MUO that achieved complete clinical and MRI-confirmed remission under a steroid-free regimen of leflunomide and mycophenolate mofetil (MMF), with sustained clinical and laboratory stability over a 2-year treatment course. Although these agents have most commonly been incorporated into MUO management as adjuncts to glucocorticoids (8–10), previous studies in other immune-mediated diseases support their immunosuppressive efficacy and acceptable safety profiles (11, 12). The immunopathogenesis of MUO is thought to involve dysregulated adaptive immune responses, including T-cell–mediated mechanisms, B-cell activation, and blood–brain barrier disruption, resulting in multifocal parenchymal and meningeal inflammation (1, 4). While glucocorticoids exert broad anti-inflammatory effects across these pathways, MMF and leflunomide provide more targeted immunomodulation by inhibiting lymphocyte proliferation and pyrimidine synthesis, respectively (11, 12). Notably, the therapeutic impact of leflunomide appears to vary depending on disease context, as it has not consistently improved outcomes when combined with glucocorticoids in other immune-mediated disorders, such as immune-mediated polyarthritis (15).

The omission of glucocorticoids at the initiation of therapy in this case was a deliberate clinical decision rather than an oversight and was guided by both patient-specific factors and the presumed disease biology of MUO. Prior to referral, the dog had already received glucocorticoid treatment at another institution and developed clinically significant adverse effects, prompting the owners to seek an alternative therapeutic approach. Given the patient’s very small body size and the anticipated need for prolonged immunosuppressive therapy, further steroid exposure was considered likely to impose a disproportionate burden of cumulative toxicity.

Importantly, MUO is increasingly regarded as a chronic immune-mediated disorder rather than a condition amenable to definitive control through short-term anti-inflammatory intervention alone. From this perspective, durable disease control is more closely aligned with sustained immunomodulation than with transient suppression of inflammation. On the basis of this pathophysiological framework, early initiation of MMF and leflunomide was selected to directly target pathogenic immune mechanisms while avoiding the long-term adverse effects associated with glucocorticoids.

Although short-term glucocorticoid therapy is commonly employed to achieve rapid clinical stabilization in severe cases of MUO, its omission in this patient did not compromise the overall trajectory of neurological recovery or the achievement of complete MRI-confirmed lesion resolution. This case therefore illustrates that, in carefully selected patients—particularly those with prior steroid intolerance or a high risk of cumulative toxicity—early steroid-free immunosuppressive therapy may represent a rational and biologically coherent treatment strategy.

Several limitations should be acknowledged. First, although MRI and CSF findings strongly supported MUO, the diagnosis remained presumptive because histopathologic confirmation was not obtained, an inherent limitation in many clinical MUO cases, particularly in toy-breed dogs. Second, as a single-case report, these findings cannot be generalized to all dogs with MUO. Third, although unlikely given the severity of the initial imaging abnormalities and the progressive clinical improvement observed following treatment, the possibility of spontaneous remission cannot be entirely excluded.

Despite these limitations, this report suggests that steroid-free immunosuppression using leflunomide and MMF may be a feasible therapeutic option when glucocorticoids are contraindicated or pose unacceptable risk, particularly in small-breed dogs requiring long-term disease control. The complete radiologic and clinical resolution observed in this dog highlights the potential role of targeted immunosuppressive strategies in achieving durable remission while minimizing the well-recognized complications associated with chronic glucocorticoid exposure. Future studies, including multi-center case series and prospective trials, will be necessary to identify patient subsets most likely to benefit from steroid-sparing approaches, refine treatment protocols, and compare outcomes directly with conventional steroid-based regimens.

## Data Availability

The raw data supporting the conclusions of this article will be made available by the authors, without undue reservation.
